# Proteome data of *Anopheles stephensi* hemolymph using high resolution mass spectrometry

**DOI:** 10.1016/j.dib.2018.04.031

**Published:** 2018-04-24

**Authors:** Gourav Dey, Ajeet Kumar Mohanty, Manish Kumar, Sreelakshmi K. Sreenivasamurthy, Ashwani Kumar, T.S. Keshava Prasad

**Affiliations:** a Center for Systems Biology and Molecular Medicine, Yenepoya Research Centre, Yenepoya (Deemed to be University), Mangalore 575018, India; b Institute of Bioinformatics, Discoverer Building, International Tech Park, Bangalore 560066, India; c Manipal Academy of Higher Education, Madhav Nagar, Manipal, Karnataka 576104, India; d National Institute of Malaria Research, Field Station, Campal, Panaji, Goa 403001, India

## Abstract

The article provides insights into the protein expression in *Anopheles stephensi* hemolymph. We carried out data acquisition using a high-resolution LTQ-Orbitrap Velos mass spectrometer to identify the hemolymph proteins of *An. stephensi*. Experimentally derived mass spectrometry data was analyzed using Proteome Discoverer 2.1 software using two different search algorithms SEQUEST and MASCOT. A total of 1091 proteins were identified from the hemolymph. The identified proteins were categorized for their role in biological processes and molecular functions. The interactions between these proteins were predicted using STRING online tool. Relation can be drawn between the data provided in this study to the already published article “Integrating transcriptomics and proteomics data for accurate assembly and annotation of genomes” (Prasad et al., 2017) [1].

**Specifications Table**

**Table:** 

Subject area	Biology
More specific subject area	Vector biology
Type of data	Excel files, figures
How data was acquired	LTQ-Orbitrap Velos ETD mass spectrometer (Thermo Scientific, Bremen, Germany)
	Proteome Discoverer 2.1 and MASCOT search engine (Matrix Science, London, UK; version 2.2)
	Protein database *Anopheles stephensi* Indian strain (www.VectorBase.org, release February 25, 2014)
Data format	Analyzed
Experimental factors	Hemolymph were collected from sugar fed mosquitoes and proteins extracted.
Experimental features	Proteome profiling of *Anopheles stephensi* hemolymph
Data source location	Goa and Bangalore, India
Data accessibility	Raw mass spectrometric data is available via a web application (ProteomeXchange) Consortium (http://proteomecentral.proteomexchange.org) via the PRIDE partner repository with the dataset identifier PXD001128.
	Analyzed data is provided along with this article as excel sheets.

**Value of the data**

• The data provides a list of proteins identified to be expressed in the adult *An. stephensi* female hemolymph.
• The data provides an insight into the class of proteins identified in the hemolymph and their associated biological processes.
• The data provides a protein-protein interaction map for *An. stephensi* hemolymph proteins.
• The data provides a platform for further experiments to understand the molecular mechanism involved in the hemolymph of *An. stephensi*.

## 1 Data

**Fig. 1:**
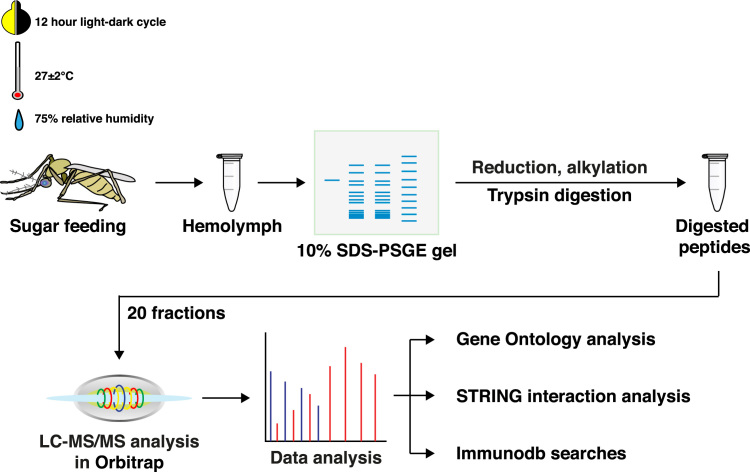
Graphical representation of mosquito insectary conditions (light-dark cycle, temperature and humidity), sample processing steps, fractionation method used, and workflow of data analysis undertaken in the study.

To identify the proteins expressed in the mosquito hemolymph, we carried out proteomic analysis of hemolymph from sugar fed female *An. stephensi* (Liston strain) mosquitoes using high-resolution mass spectrometer. Proteins extracted from the samples were fractionated at protein level using 10% SDS-PAGE. Each fraction was then analyzed separately, which, resulted in the identification of 55,204 peptide-spectrum matches (PSMs) corresponding to 8999 peptide groups and 1091 proteins, of which, multiple PSMs were observed for 1057 proteins. The experimental workflow is depicted in (Fig. 1) and the complete list of identified proteins and their corresponding peptides have been provided in Supplementary Table S1 and S2.

## 2 Experimental design, materials and methods

### 2.1 Maintenance of mosquito colony

Female An. stephensi mosquitoes were obtained from the insectary at National Institute of Malaria Research, Field Station at Goa, where a continuous cyclic colony of the species is being maintained at 27 ± 2 °C, 75% relative humidity, a cycle of 12 h in light and 12 h in darkness. The adult mosquitoes were maintained on 10% glucose solution soaked in a cotton pad. From these cyclic colonies, female mosquitoes were used for the experiments and hemolymph was extracted as described [1].

### 2.2 Protein extraction

Mosquito heads were decapitated, and the thorax gently pressed to discharge out the hemolymph which, was then collected and stored at − 80 °C until further use. Samples were homogenized in 2% SDS using a probe sonicator. The tissue lysate was centrifuged at 14,000 rpm for 10 min at 4 °C and the supernatant collected. The extracted proteins were quantified according to modified Lowry's method (Bio-Rad DC Protein assay) and normalized on 10% SDS-PAGE.

### 2.3 Fractionation

For in-gel digestion, 300 µg of protein was resolved on a 10% SDS-PAGE gel as discussed previously [1], [2], [3]. Gel bands were excised out of the gel based on their intensity and then destained, reduced and alkylated priory to trypsin digestion. Trypsin digestion was carried out at 37 °C overnight using Promega sequencing grade trypsin. Digested peptides were extracted out using 40% acetonitrile solution in 0.1% of formic acid. Extracted peptides were desalted using stage-tip C_18_ columns and vacuum dried prior to acquisition on mass spectrometer.

### 2.4 Mass spectrometry analysis

The fractions were analyzed on LTQ-Orbitrap Velos mass spectrometer (Thermo Scientific, Bremen, Germany) interfaced with Easy-nLCII (Thermo Scientific, Bremen, Germany). Peptides were initially enriched on a reversed phase liquid chromatography (RPLC) pre-column (2 cm, 5 μ–100 Ǻ), followed by separation on an analytical column (11 cm, 3 μ–100 Ǻ) packed in-house with magic AQ C_18_ material (Michrom Bioresources, Inc, Auburn, CA). The solvent system used included 0.1% aqueous formic acid as solvent A and 95% acetonitrile, 0.1% formic acid as solvent B. The peptides were loaded on the trap column using solvent A, followed by resolution on the analytical column using a gradient of 10–35% solvent B for 75 min at a constant flow rate of 0.25 μL/min. The spray voltage and heated capillary temperature were set to 2.0 kV and 220 °C, respectively and data was acquired in a data dependent manner. Fifteen most intense precursor ions were selected for fragmentation from each MS scan. MS and MS/MS scans were acquired in an Orbitrap mass analyzer with mass resolution of 30,000 and 15,000 at 400 *m/z*, respectively. The peptides were fragmented by higher energy collision dissociation with normalized collision energy of 39%. The automatic gain control (AGC) for full FTMS was set to 1 million ions and for FT MS/MS was set to 0.1 million ions with maximum accumulation time of 100 ms and 200 ms, respectively.

### 2.5 Gene Ontology categorization and generation of interaction map

Categorization of the identified proteins was performed by fetching information provided in the Panther database [4]. As Panther database has identifiers for the much-studied *An. gambiae* but not the *An. stephensi* proteins. We used Biomart (version 0.7) [5] tool provided through VectorBase [6] to fetch the corresponding *An. gambiae* orthologs for the *An. stephensi* proteins (Supplementary Table S3). These *An. gambiae* identifiers were then used to fetch the Gene Ontology related information.

To generate a protein-protein interaction map of the identified proteins, we used STRING (Search Tool for the Retrieval Interacting Genes/Proteins) online tool (version 10.5).plugin (version 1.1.0) [7].

### 2.6 Data analysis

The data was searched against the protein database of *An. stephensi* proteins using Proteome Discoverer software, version 2.1 (Thermo Fischer Scientific, Bremen, Germany). The processing workflow consisted of spectrum selector and percolator besides SEQUEST and Mascot search nodes. Searches were initiated with trypsin as enzyme, allowing a single missed cleavage with a minimum peptide length of 6 amino acids. Other parameters include carbamidomethylation of cysteine as static modifications and oxidation of methionine as variable modification. A false discovery rate (FDR) of 1% was applied to the results.

**Fig. 2:**
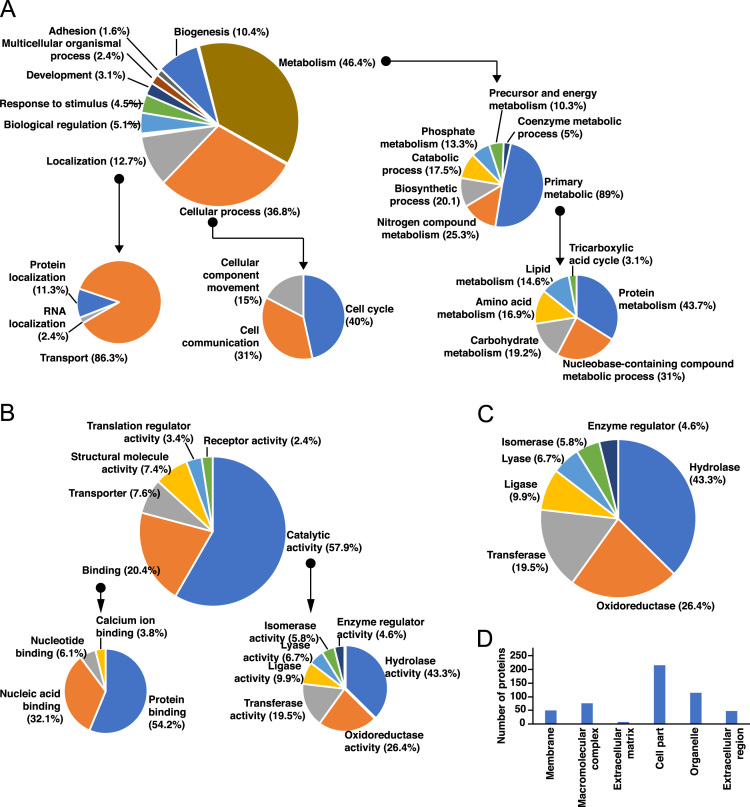
Functional analysis of the identified protein in hemolymph was performed using PANTHER database. Pie chart representation of A) Biological processes, B) Molecular function, C) Protein Class and Bar graph representation of D) Sub-cellular localization. Major processes and functions identified were further analyzed for their sub-class categorization.

The proteins identified from these searches were analyzed further for functional categorization based on biological processes, molecular function, protein class and cellular component using Gene Ontology annotations provided in Panther database. Majority of the identified proteins were found to be associated with metabolism (46.4%), cellular processes (36.8%) and localization (12.7%). The detailed Gene Ontology categorization of the identified proteins is provided in Fig. 2.

**Fig. 3:**
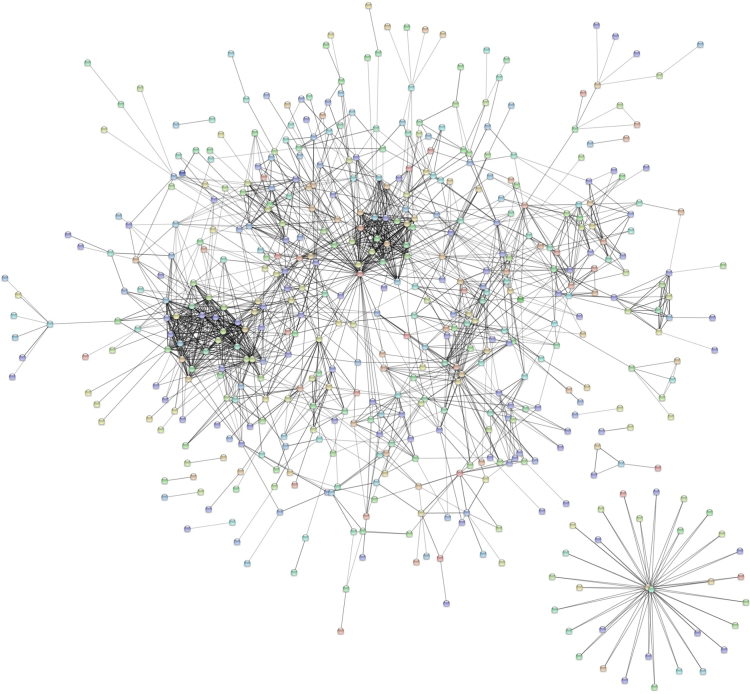
Predicted protein-protein interaction map of proteins with few distinct clusters, identified in the *An. stephensi* hemolymph. The interaction map was generated using STRING online tool with default parameters.

**Fig. 4:**
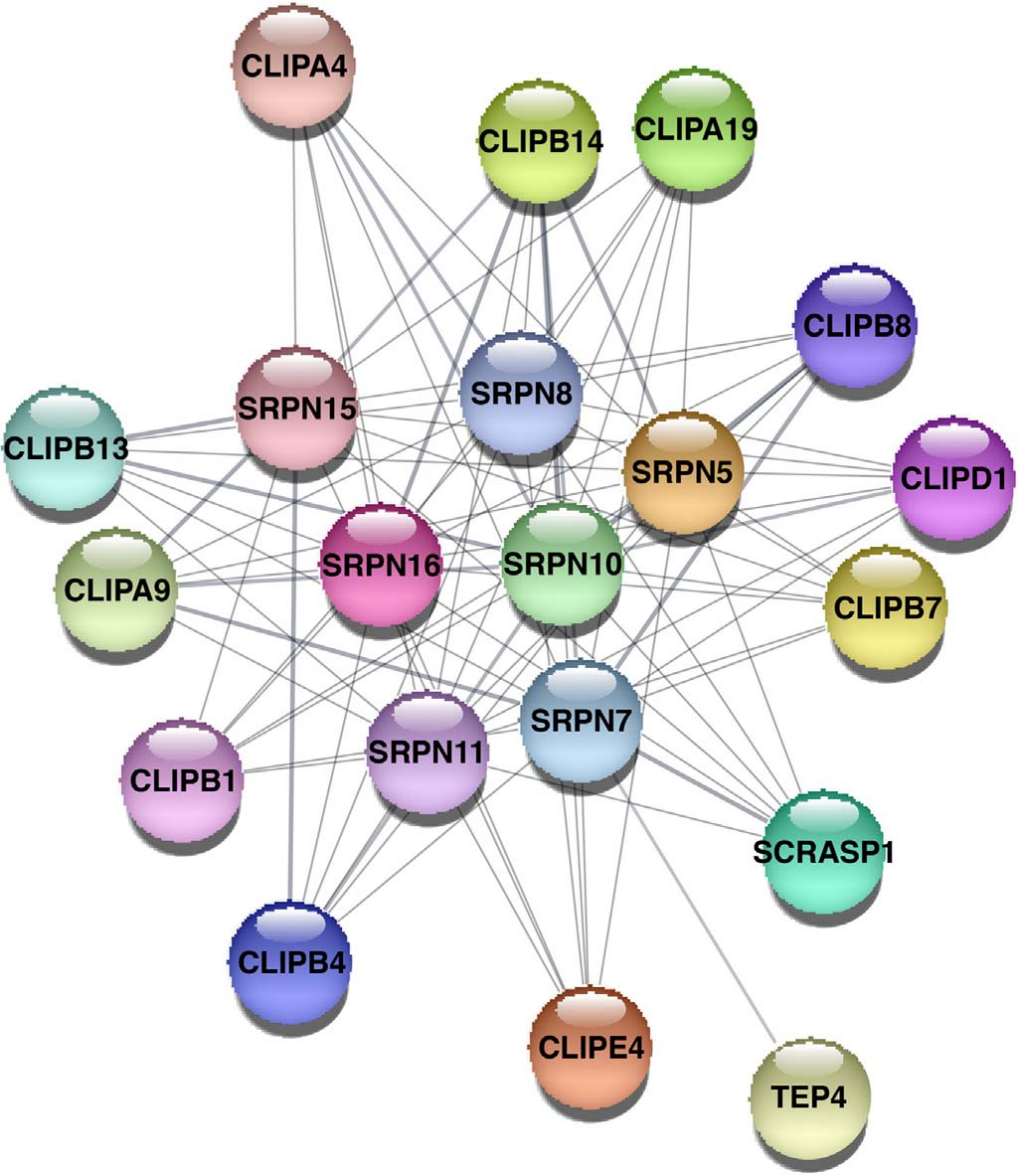
Predicted protein-protein interaction map of proteins identified in hemolymph and having a potential role in immunity (predicted by mapping to ImmunoDB database). Online STRING tool with default parameters was used to generate the represented interaction map.

The total identified proteins were analyzed using online STRING tool to generate an interacting map for the hemolymph proteins (Fig. 3, Supplementary Table S4). We also, compared the list of total identified proteins with the list of immune related proteins provided in ImmunoDB. A total of 40 proteins identified in the hemolymph were found to be associated with immune related functions (Supplementary Table S5) of which, 20 proteins were predicted to interact with each other (Fig. 4, Supplementary Table S6).

## Appendix A Supplementary material

Supplementary Table S1: List of proteins identified in hemolymph of female *An. stephensi* using LC-MS/MS. Supplementary Table S2: List of peptides identified by LC-MS/MS analysis in hemolymph of female *An. stephensi*. Supplementary Table S3: List of *An. stephensi* (Indian strain) hemolymph proteins along with their orthologs in *An. stephensi* (SDA-500 strain) and *An. gambiae*. Supplementary Table S4: List of hemolymph proteins in *An. stephensi* predicted to be interacting among each other. Supplementary Table S5: List of hemolymph proteins in *An. stephensi* predicted to be associated with immune related functions using data provided through Immunodb. Supplementary Table S6: List of hemolymph proteins in *An. stephensi* predicted to be associated with immune related functions and interacting with each other.
