# Supplementary material for: Proteome data of Anopheles stephensi hemolymph using high resolution mass spectrometry
Source: Data Brief. 2018 Apr 24;18:1441–7. doi: 10.1016/j.dib.2018.04.031 (PMC5997892; doi:10.1016/j.dib.2018.04.031)
Supplement: Supplementary file 1 — Transparency document [file mmc1.zip › Conflicts of Interest.pdf]

Conflicts of Interest

The authors declare that they have no conflict of interest.
